# Detection of Water Holdup in Oil–Water Flows Using a Curved Microstrip Sensor with Resonance-Enhanced Response

**DOI:** 10.3390/s26134060

**Published:** 2026-06-26

**Authors:** Gaoyang Zhu, Yunjun Zhang, Junlin Feng, Xinhua Sun, Shucheng Liang, Bin Wang, Muzhi Gao

**Affiliations:** 1 College of Electronic and Information Engineering, Shandong University of Science and Technology, Qingdao 266590, China; gaoyangzhu@sdust.edu.cn (G.Z.); 202483130144@sdust.edu.cn (Y.Z.); 202483130080@sdust.edu.cn (J.F.); 202583130103@sdust.edu.cn (X.S.); 202582130032@sdust.edu.cn (S.L.); 2 College of Control Science and Engineering, China University of Petroleum (East China), Qingdao 266580, China; wangbin2015@upc.edu.cn

**Keywords:** water holdup, curved microstrip transmission line sensor, oil–water flow, resonance-based sensing, dielectric characterization

## Abstract

**Highlights:**

**What are the main findings?**

- A curved microstrip transmission line sensor was proposed for water holdup detection in oil–water flows.
- Experimentally measured dielectric properties were incorporated into full-wave simulations to improve physical consistency.
- Resonance frequency was found to be a reliable feature for stratified flow, especially when magnitude and phase responses have reduced resolution.

**What are the implications of the main findings?**

- The proposed sensor provides a feasible approach for non-invasive water holdup detection in oil–water flows.
- Using measured dielectric properties improves the reliability of simulation-based sensor modeling.
- Resonance frequency can be used as a robust sensing feature to improve detection under stratified flow conditions.

**Abstract:**

Accurate water holdup measurement in oil–water flows remains challenging due to flow-regime-dependent dielectric distributions and the limited sensitivity of conventional amplitude- or phase-based sensing features. This paper proposes a curved microstrip transmission-line sensor that jointly exploits broadband scattering responses and resonance-frequency shifts to characterize water holdup. The curved geometry increases the effective electrical length within a compact footprint, strengthens field interaction with the surrounding medium, and introduces resonance behavior within the operating band. To improve the physical consistency of numerical modeling, the frequency-dependent complex permittivity of oil–water mixtures is experimentally measured using an open-ended coaxial probe and directly incorporated into full-wave electromagnetic simulations. Both emulsion and stratified oil–water conditions are investigated through simulation and experimental validation. The results show that, under emulsion conditions, the magnitude and phase of S_11_ and S_21_ exhibit clear monotonic responses to water holdup. Under stratified conditions, conventional magnitude and phase features exhibit reduced resolution due to the spatially non-uniform dielectric distribution. In this case, variations in water holdup primarily modify the interface position rather than the overall dielectric volume, resulting in relatively small perturbations to the effective permittivity experienced by the guided electromagnetic field. Nevertheless, the resonance frequency remains highly sensitive and shifts monotonically with water holdup. The proposed sensor combines a resonant frequency with broadband magnitude and phase responses, where the resonant frequency provides a stable and reliable indicator across different flow conditions. The results demonstrate the potential of curved microstrip transmission-line structures for compact and reliable water holdup measurement in complex oil–water flow environments.

## 1. Introduction

Water holdup, defined as the volumetric fraction of water within an oil–water flow system, is a critical parameter for characterizing oil–water two-phase flows and plays an essential role in petroleum production, pipeline transportation, and reservoir management. Accurate water holdup measurement is essential for evaluating production conditions, optimizing reservoir management, and ensuring safe operation under complex flow environments [1]. However, reliable water holdup detection remains challenging due to the heterogeneous distribution of oil and water phases and variations in flow regimes [2].

Traditional water holdup detection methods mainly include capacitance-based, conductance-based, and nuclear measurement techniques. Capacitance sensors exploit the contrast in dielectric constants between oil and water, while conductance sensors rely on the difference in conductivity between the two phases [3,4,5,6]. Although these methods have been widely applied in practice, their performance is often limited to specific water holdup ranges and is highly sensitive to variations in flow patterns. Nuclear-based techniques can provide full-range measurements but suffer from high cost, safety concerns, and complex system requirements [7]. Consequently, there is a strong demand for alternative sensing approaches that can achieve wide-range, high-resolution, and robust water holdup detection [8].

In recent years, microwave electromagnetic sensing techniques, particularly those based on transmission-line structures, have attracted increasing attention for multiphase flow detection [9]. When an oil–water mixture is loaded around a microstrip transmission line, changes in water holdup modify the effective dielectric properties of the surrounding medium, thereby affecting the propagation characteristics of electromagnetic waves [10]. By analyzing scattering parameters, particularly the magnitude and phase responses, transmission-line sensors provide continuous characterization of water holdup through their sensitivity to dielectric variations. Several microwave and transmission-line sensing structures have been investigated for oil–water measurement. Wei et al. developed a coplanar microstrip transmission-line sensor for water holdup measurement in oil–water two-phase flows, demonstrating that transmission-line scattering responses can effectively reflect changes in water content [11]. Zhang et al. proposed an embedded serpentine microstrip line array for monitoring the oil–water separation process, in which vertically and horizontally arranged sensing elements were used to distinguish different flow states based on the phase response of S_21_ [12]. Sattari and Hayati developed an ANN-assisted microstrip sensor for oil–water volume fraction estimation [13], while Dehkalani et al. reported a dual-band microstrip sensor optimized with an RBF neural network for oil–water mixture analysis [14]. Resonant microwave structures have also been explored; for example, Su et al. used a complementary split-ring resonator (CSRR) to estimate the complex permittivity of oil samples [15]. For downhole applications, Shen et al. proposed a wideband microwave sensing method for water-cut monitoring in pipelines [16]. In addition, Wei et al. developed transmission-line-based water holdup sensors and sensor arrays for oil–water two-phase flow measurement, demonstrating the feasibility of using distributed electromagnetic responses to detect water holdup [17]. These studies confirm that microwave and transmission-line sensing techniques are promising for oil–water flow characterization.

However, existing methods still present several limitations. First, many transmission-line sensors mainly utilize a single feature, such as unwrapping phase, attenuation, or local resonance response, which may limit measurement robustness when the flow regime changes [18]. Second, resonant structures, including split-ring resonator (SRR) and CSRR sensors, can provide high sensitivity, but their sensing volume and applicable flow configurations are often limited, making them less suitable for complex oil–water distributions such as stratified flow [19]. Third, compact transmission-line sensors are usually constrained by limited electrical length, which weakens phase accumulation and reduces resonance sensitivity, especially when low-frequency and low-loss operation is desired. Moreover, many numerical studies describe oil–water mixtures using theoretical dielectric mixing models, such as Maxwell–Garnett or Bruggeman formulations. Although these models are useful for approximate analysis, they may not fully capture the actual frequency dispersion and dielectric loss of real oil–water emulsions, leading to uncertainties in simulated sensor responses.

To address these limitations, this paper proposes a curved microstrip transmission-line sensor for water holdup detection in oil–water flows by exploiting both its propagation characteristics and resonant behavior. First, a curved microstrip transmission line structure is designed to introduce resonance behavior while maintaining compactness and practical waterproofing capability. Second, the frequency-dependent complex permittivity of oil–water mixtures is experimentally measured using an open-ended coaxial probe and directly incorporated into full-wave electromagnetic simulations, improving the physical consistency of the numerical model. Third, the sensing performance is systematically investigated under both emulsion and stratified oil–water conditions. The results show that magnitude, phase, and resonance features provide mutually consistent information under emulsion conditions, while resonance frequency remains highly sensitive under stratified conditions where conventional magnitude and phase features exhibit reduced resolution. The proposed approach offers a practical, scalable solution for accurate water holdup detection and has potential applications in oilfield production monitoring and related multiphase flow measurement scenarios.

## 2. Theory of the System

### 2.1. Curved Microstrip Transmission Line Sensor Design

The proposed sensor utilizes a curved microstrip transmission line configuration to overcome the inherent limitations of conventional straight microstrip transmission line sensors with respect to sensitivity, resonance controllability, and spatial efficiency. In practical oil–water flow monitoring applications, the available installation space is often limited, while high sensitivity to dielectric variations is required. The curved microstrip transmission line geometry provides an effective solution, increasing the electrical length of the guided path without proportionally enlarging the sensor’s physical footprint [20].

**Figure 1:**
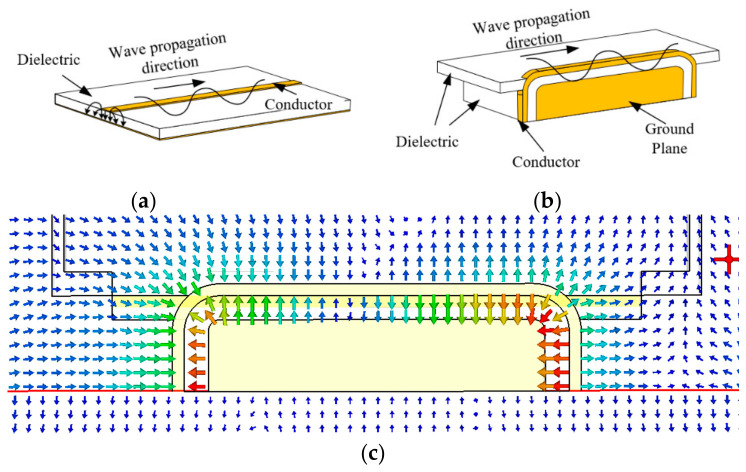
Schematic illustration of the (**a**) straight, (**b**) curved microstrip transmission lines, (**c**) electric-field distribution. In (**c**), the arrows indicate the direction of the electric field, and the colors represent the field intensity, decreasing from red to blue.

A conventional microstrip transmission line is schematically illustrated in Figure 1a, where the effective electrical length is strictly constrained by the physical size of the structure. As a result, achieving low resonance frequencies or enhanced phase sensitivity typically requires a longer straight line, which is undesirable in compact sensing environments [21]. To address this issue, a curved microstrip transmission line sensor is proposed in this work. The overall configuration and geometry of the sensor are shown in Figure 1b.

Compared with a traditional straight microstrip transmission line, the curved transmission-line structure offers several distinct advantages. First, the curved geometry effectively increases the electrical length *L* of the microstrip transmission line within a limited physical footprint. According to the half-wavelength resonance condition,

(1) $$f_{r}=\frac{c}{2L\sqrt{\varepsilon_{eff}}}$$

where $f_{r}$ is the resonance frequency, *c* is the speed of light in free space, and $\varepsilon_{eff}$ is the effective permittivity. The resonance frequency is inversely proportional to the electrical length. By increasing *L* via curvature, the resonant frequency can be shifted to a lower band without increasing the sensor size. Operating at lower frequencies is beneficial for practical implementation, as radio-frequency components are generally less expensive and less demanding in terms of fabrication tolerance. Moreover, for high-water-content media with large dielectric constants, high-frequency losses are significantly higher than those at lower frequencies [22]; therefore, operating at lower frequencies provides improved penetration and more stable resonance features.

Second, as shown in Figure 1c, the curved microstrip transmission line introduces geometric discontinuities at the bending regions. These discontinuities modify the electromagnetic field distribution along the line, thereby promoting stronger field exposure near the sensing region. Since the effective dielectric constant of a microstrip transmission line is fundamentally determined by the spatial distribution of electric-field energy, this structural feature is favorable for enhancing the interaction between the guided fields and the surrounding oil–water medium.

Finally, from an engineering perspective, the compact, continuous routing of the curved microstrip transmission line improves the sensor’s mechanical stability. The structure exhibits enhanced resistance to vibration, mechanical stress, and long-term operation in liquid or high-humidity environments. Moreover, the curved layout facilitates centralized design of the sensing region, which is advantageous for waterproof encapsulation and packaging. These characteristics make the proposed structure well-suited for practical deployment and scalable fabrication.

**Figure 2:**
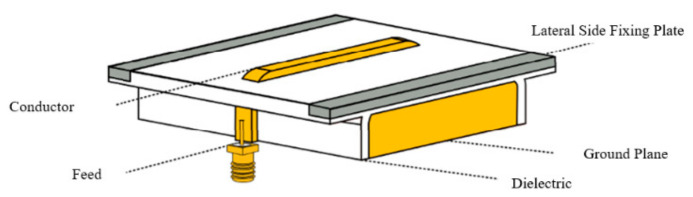
Overall configuration of the proposed curved transmission-line sensor.

As illustrated in Figure 2, the sensor consists of four main components: a ground plane, a microstrip line, a substrate, and SMA connectors. The ground plane is fabricated from copper and is positioned beneath the substrate to provide a stable reference conductor and mechanical support. The microstrip line is located on the upper surface of the substrate and consists of a flat copper conductor coated with polyimide insulation. The polyimide layer provides electrical insulation as well as resistance to oil, moisture, and elevated temperatures. In the present prototype, the thickness of the polyimide protective layer is 0.03 mm. This layer is mainly used to protect the metallic conductor from oxidation, corrosion, impact, and wear in the oil–water environment. The thickness of the protective layer can influence the sensor response because a thicker layer increases the distance between the microstrip conductor and the tested oil–water medium, thereby weakening the electromagnetic coupling. In contrast, an excessively thin layer may reduce mechanical protection. Therefore, the 0.03 mm-thick polyimide layer was selected to provide necessary protection while keeping its influence on the sensor response relatively limited. To realize the curved transmission path, the microstrip line passes through a dedicated opening in the substrate and is bent to a controlled radius of curvature. SMA connectors are soldered at both ends of the microstrip line to provide reliable electrical interfacing with external measurement instruments.

### 2.2. Equivalent Three-Section Transmission-Line Circuit Model

**Figure 3:**
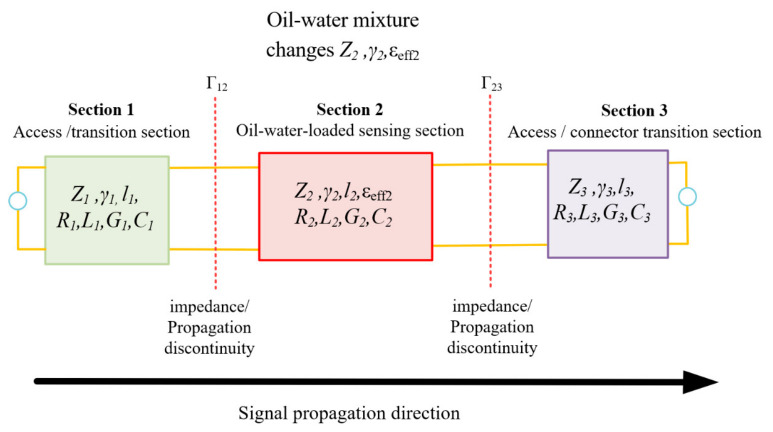
Equivalent three-section transmission-line circuit model of the proposed curved microstrip sensor.

To further clarify the physical origin of the resonance response, the proposed curved microstrip sensor can be interpreted using an equivalent three-section transmission-line circuit model, as shown in Figure 3. Along the signal propagation direction, the sensor is approximately divided into three cascaded transmission-line sections. Section 1 and Section 3 represent the input/output access and connector transition regions that are not directly loaded by the oil–water mixture, whereas Section 2 represents the main sensing region electromagnetically coupled with the material under test. Each section can be described by distributed transmission-line parameters $R_{i}$, $L_{i}$, $G_{i}$, and $C_{i}$, or equivalently by its characteristic impedance $Z_{i}$, propagation constant $\gamma_{i}$, and physical length $L_{i}$.

For the i-th transmission-line section, the characteristic impedance and propagation constant can be expressed as

(2) $$Z_{i}=\sqrt{\frac{R_{i}+j\omega L_{i}}{G_{i}+j\omega C_{i}}}$$

(3) $$\gamma_{i}=\sqrt{\left(R_{i}+j\omega L_{i})(G_{i}+j\omega C_{i}\right)}=\alpha_{i}+j\beta_{i}$$

where $R_{i}$, $L_{i}$, $G_{i}$, and $C_{i}$ are the per-unit-length resistance, inductance, conductance, and capacitance of the i-th section, respectively. $Z_{i}$ is the characteristic impedance, $\gamma_{i}$ is the propagation constant, $\alpha_{i}$ is the attenuation constant, and $\beta_{i}$ is the phase constant.

The oil–water mixture mainly affects Section 2 by changing its effective dielectric constant, dielectric loss, characteristic impedance, attenuation constant, and phase constant. Because the three sections have different characteristic impedances and propagation constants, discontinuities are formed at the interfaces between adjacent sections. The corresponding reflection coefficients can be approximately written as $\Gamma_{12}$.

(4) $$\Gamma_{12}=\frac{Z_{2}-Z_{1}}{Z_{2}+Z_{1}},\;\Gamma_{23}=\frac{Z_{3}-Z_{2}}{Z_{3}+Z_{2}}$$

The overall transmission response of the sensor can be described by cascading the transmission matrices of the three sections:

M = M_1_M_2_M_3_ (5)

Therefore, variations in water holdup mainly modify $\varepsilon_{\mathrm{eff}2}$, $Z_{2}$, and $\gamma_{2}$ of the oil–water-loaded sensing section, which further changes the interface reflection, attenuation, phase condition, and resonance frequency of the S-parameter response.

### 2.3. Sensing Principle

The sensing mechanism of the proposed curved transmission-line sensor relies on the dependence of electromagnetic wave propagation on the dielectric properties of the surrounding medium. When oil–water mixtures are loaded into the sensing region, variations in water holdup lead to systematic changes in the effective permittivity and loss of the medium. These changes modulate the propagation constant and impedance characteristics of the microstrip transmission line, resulting in measurable variations in both the magnitude and phase of the scattering parameters [23,24]. By jointly exploiting these variations, a multi-information sensing framework for water holdup detection is established.

For a general microstrip transmission line, the propagation constant can be expressed as

(6) γ=α+jβ

where α is the attenuation constant and β is the phase constant. In quasi-TEM transmission, β is primarily governed by the effective permittivity $\varepsilon_{eff}$.

(7) $$\beta =\frac{\omega }{c}\sqrt{\varepsilon_{eff}}$$

where ω is the angular frequency and c is the speed of light in free space. As water exhibits a much higher permittivity than oil, an increase in water holdup leads to an increase in $\varepsilon_{eff}$, which in turn produces a larger phase constant. Meanwhile, the higher dielectric loss associated with water-rich mixtures increases the attenuation constant α, resulting in stronger signal attenuation along the propagation path.

The magnitude of the reflection coefficient S_11_ reflects the impedance mismatch between the input impedance of the sensor structure and the reference impedance *Z*_0_ of 50 Ω:

(8) $$S_{11}=\frac{Z_{in}-Z_{0}}{Z_{in}+Z_{0}}$$

where $Z_{in}=R+jX$. When the surrounding dielectric changes, the electromagnetic field distribution around the microstrip line is modified, leading to an increase in the equivalent capacitance of the structure [25,26,27]. For the capacitive-dominated case, the reactance can be approximated as

(9) $$X\approx -\frac{1}{\omega C}$$

As $\varepsilon_{eff}$ increases, the equivalent capacitance C increases, and the input reactance becomes more capacitive, resulting in increased reflection magnitude.

The magnitude of the transmission coefficient *S*_21_ is mainly influenced by propagation attenuation. Higher permittivity and the accompanying dielectric loss increase the effective attenuation constant, resulting in greater transmission loss [28]. Consequently, the magnitude of *S*_21_ typically decreases as the dielectric constant of the surrounding medium increases, providing a monotonic, physically interpretable indicator for characterizing water holdup.

In addition to magnitude features, phase information provides a highly sensitive dimension for sensing. The phase of *S*_21_ can be expressed as

(10) $$\phi_{21}=-\beta L$$

indicating that phase accumulation increases with both effective permittivity and electrical length. This directly explains why extending the electrical length through a curved geometry enhances phase sensitivity.

Beyond broadband magnitude and phase responses, resonance behavior provides an additional frequency-domain feature. For half-wavelength resonance,

(11) $$f_{r}=\frac{c}{2L\sqrt{\varepsilon_{eff}}}$$

which shows that increasing $\varepsilon_{eff}$ leads to a downward shift in resonance frequency.

Therefore, water holdup can be characterized using three complementary observables: magnitude, phase, and resonance frequency. Among these, the resonant frequency is particularly sensitive to small dielectric variations, as it depends on the accumulated phase along the entire transmission path rather than on local field variations.

### 2.4. Sensor Geometry Design

Based on the sensing mechanisms, the geometric parameters of the curved microstrip transmission line sensor are designed to balance resonance frequency positioning, impedance matching, sensitivity, and fabrication feasibility. The key parameters include the effective transmission length *L_s_*, the microstrip width *W_a_*, ground-related dimensions (*W_b_*, *W_s_*), the substrate thicknesses (*H_a_*, *H_b_*, *H_s_*, *H_g_*), and the curvature geometry.

Variations in the effective length and curvature directly influence the resonance frequency, while the microstrip width and substrate thickness determine impedance matching and field confinement. Increasing L_s_ shifts the resonance toward lower frequencies, which is beneficial for reducing dielectric loss in high-water-content conditions. However, excessive curvature or deviations in width may deteriorate impedance matching and increase insertion loss. Therefore, the final dimensions are selected to ensure stable resonant behavior, acceptable impedance matching near 50 Ω, and sufficient sensitivity to dielectric perturbations.

**Figure 4:**
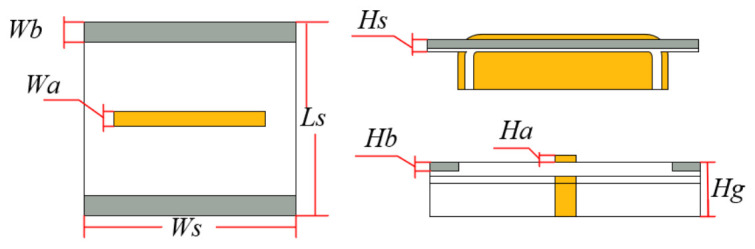
Definition of geometrical parameters of the proposed sensor.

Figure 4 illustrates the final dimensional layout of the proposed curved microstrip transmission line sensor. The corresponding numerical values are summarized in Table 1. The selected dimensions provide a balanced trade-off among sensitivity, resonance positioning, mechanical robustness, and fabrication feasibility, forming a solid foundation for the numerical simulations and experimental investigations presented in the following sections.

## 3. Numerical Modeling and Simulation Analysis

### 3.1. Broadband Dielectric Measurement of Oil–Water Mixtures

To ensure that subsequent numerical simulations accurately represent the real electromagnetic behavior of oil–water mixtures, broadband dielectric characterization is first performed experimentally. Instead of relying on theoretical effective-medium models, the complex permittivity of oil–water mixtures with varying water holdup levels is measured directly over the frequency range 1 MHz to 3 GHz.

**Figure 5:**
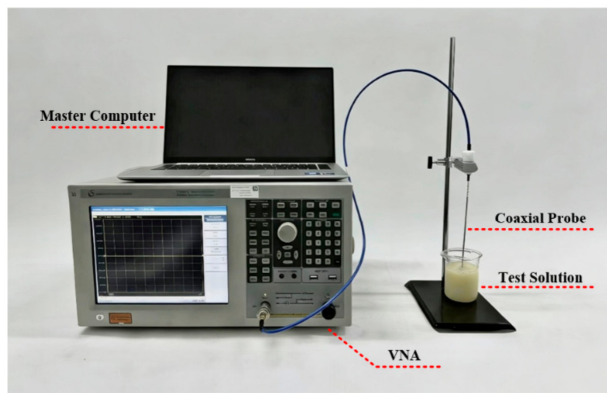
Photograph of the coaxial probe dielectric measurement setup.

The dielectric measurements are conducted using an open-ended coaxial probe connected to a vector network analyzer (VNA, T5230A, TRANSCOM, Shanghai, China), as shown in Figure 5. Prior to data acquisition, the VNA and coaxial cable are calibrated using a standard calibration kit, which includes open, short, and load standards, to eliminate systematic errors associated with the measurement instrument and the transmission path. Subsequently, the coaxial probe itself is calibrated through a custom-developed algorithm. This probe calibration procedure involves measuring the reflection coefficients of three reference media—deionized water, a short-circuit load, and air—and constructing compensation equations to correct for probe-specific and residual system errors. Through this two-stage calibration process, accurate reflection coefficients at the probe–sample interface are obtained, which serve as the basis for dielectric inversion and subsequent extraction of the complex permittivity of the oil–water mixtures.

Oil–water mixtures with water holdup ranging from 0 to 100% are prepared by mixing oil and water in controlled proportions, then adding an emulsifier and continuously stirring with a magnetic stirrer to ensure homogeneous emulsification. During measurement, the coaxial probe is vertically immersed into the mixture, with the probe tip positioned near the center of the liquid volume to minimize boundary effects. The VNA records the complex reflection coefficient over the full frequency band, and the measured data are processed using a custom-developed inversion algorithm to extract the real and imaginary parts of the complex permittivity. To ensure measurement reliability and accuracy, each test is repeated three times under identical conditions, and the final dielectric parameters are obtained by averaging the repeated measurements.

**Figure 6:**
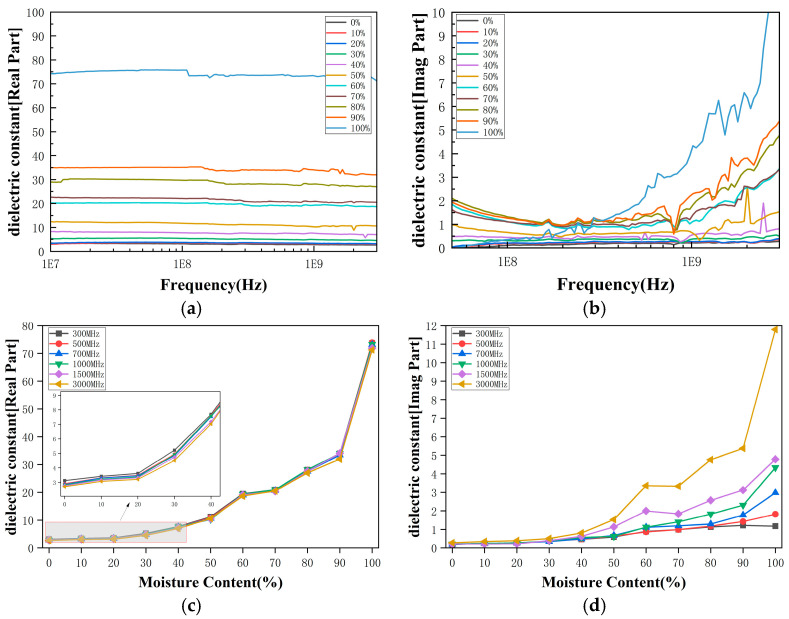
(**a**) measured real part of permittivity versus frequency for different water holdups; (**b**) measured dielectric loss versus frequency, and variation in dielectric constant with water holdup at multiple representative frequencies (**c**) real part, (**d**) image part.

Figure 6a presents the measured real part of the effective permittivity as a function of frequency for different water holdup levels. A clear, monotonic increase in permittivity is observed with increasing water content across the entire frequency band, reflecting the strong dielectric contrast between oil and water. The distinct separation among curves corresponding to different water holdups demonstrates that the dielectric response provides sufficient sensitivity for electromagnetic sensing applications. Figure 6b shows the corresponding dielectric loss factor, which exhibits stronger frequency dependence, particularly at high water holdup levels. This behavior highlights the conductive and dispersive nature of water-rich mixtures and underscores the limitations of simplified dielectric mixing models. As shown in Figure 6c,d, both the real and imaginary parts of the permittivity exhibit consistent and monotonic variation with water holdup at all selected frequencies. The experimentally measured, frequency-dependent dielectric parameters are subsequently imported into the electromagnetic simulation model as material definitions.

To further parameterize the measured dielectric spectra, the Cole–Cole model was used to fit the complex permittivity of the oil–water mixtures at different water holdups. The model is expressed as

(12) $$\varepsilon^{*}(\omega )=\varepsilon_{\infty }+\frac{\varepsilon_{s}-\varepsilon_{\infty }}{1+{\left(j\omega \tau \right)}^{1-\alpha }}+\frac{\sigma }{j\omega \varepsilon_{0}}$$

where $\varepsilon_{s}$ is the static permittivity, $\varepsilon_{\infty }$ is the high-frequency permittivity, τ is the relaxation time, α is the relaxation distribution factor, σ is the effective conductivity, and $\varepsilon_{0}$ is the vacuum permittivity. The measured real part $\varepsilon^{\prime }$ and imaginary part $\varepsilon^{\prime \prime }$ of the complex permittivity were fitted simultaneously, and the extracted parameters are summarized in Table 2.

As shown in Table 2, the fitted static permittivity $\varepsilon_{s}$ generally increases with water holdup, indicating that the increase in water content enhances the effective dielectric polarization of the oil–water mixture. The fitted relaxation time τ remains on the order of 10^−12^–10^−11^ s, reflecting the broadband dielectric dispersion behavior in the measured frequency range. It should be noted that the Cole–Cole parameters are used here mainly to provide a compact parameterized description of the measured dielectric spectra for electromagnetic modeling. Because the fitting was performed within the finite measured frequency range, some parameters, especially $\varepsilon_{\infty }$, α, and σ, should be interpreted as effective fitting parameters rather than unique material constants. These results provide a material-parameter basis for subsequent electromagnetic simulations and help explain the observed variation of sensor responses with water holdup.

### 3.2. Design and Simulation of Measurement System

Based on the experimentally measured dielectric properties, numerical models are established to investigate the electromagnetic response of the proposed curved microstrip transmission-line sensor under different oil–water flow conditions. The full-wave electromagnetic simulations were performed using CST Studio Suite(v2022). Both emulsion oil–water mixtures and stratified oil–water flow are considered, representing homogeneous and non-homogeneous dielectric distributions, respectively. The analysis focuses on the broadband magnitude and phase responses of the scattering parameters, as well as on resonance behavior, with particular emphasis on their sensitivity to water holdup.

In the present study, the investigated cases are limited to static laboratory-scale oil–water distributions, including a macroscopically homogeneous emulsified state and a two-layer stratified state. For the emulsified case, the mixture is treated as an effective homogeneous dielectric medium using the measured frequency-dependent complex permittivity, and microscopic droplets are not individually resolved. For the stratified case, the main inhomogeneity is the oil–water interface, which is explicitly represented by changing the layer thickness and interface position. More complex flow structures, such as wavy interfaces, local emulsification, droplet entrainment, slug-like distributions, and dynamic flow-pattern transitions, are beyond the scope of the present feasibility-oriented study.

**Figure 7:**
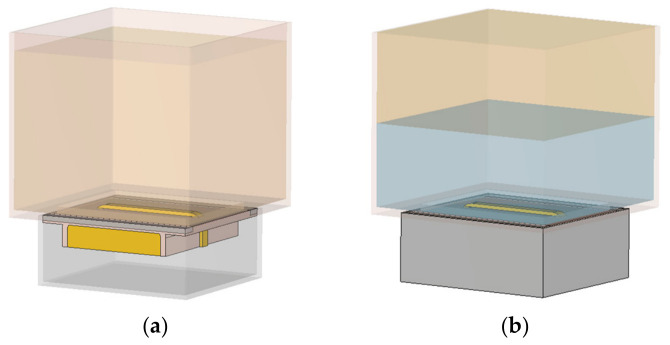
Simulation models of the curved transmission line sensor under (**a**) emulsified oil–water and (**b**) stratified oil–water conditions.

(1) **Simulation Results for Emulsion Oil–Water Mixtures**. For oil–water emulsion mixtures, the sensing region is modeled as a homogeneous effective medium whose complex permittivity matches the measured dielectric properties across different water holdup levels. The simulation configuration is illustrated in Figure 7a, in which the liquid region above the curved microstrip transmission line is modeled as a uniform dielectric volume.

**Figure 8:**
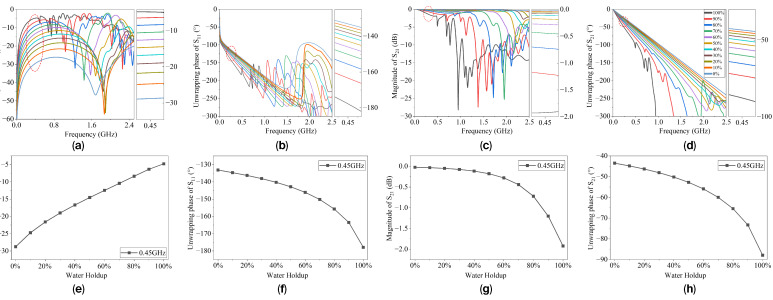
Simulated broadband responses of the sensor under emulsion conditions: (**a**) magnitude of S11, (**b**) unwrapping phase of S11, (**c**) magnitude of S21, and (**d**) unwrapping phase of S21; and single-frequency features under emulsion conditions: (**e**) magnitude of S11, (**f**) unwrapping phase of S11, (**g**) magnitude of S21, and (**h**) unwrapping phase of S21. The side panels are enlarged views of the regions marked by the red circles.

Figure 8a–d presents the simulated broadband responses of the reflection coefficient S_11_ and transmission coefficient S_21_ for emulsion oil–water mixtures. As the water holdup increases, clear, systematic variations are observed in both the magnitude and phase responses across the entire frequency band. The magnitude of S_11_ increases gradually with water holdup, indicating an enhanced impedance mismatch due to increased effective permittivity and equivalent capacitance of the structure. Meanwhile, the magnitude of S_21_ decreases monotonically with increasing water content, reflecting greater propagation attenuation due to higher dielectric loss in water-rich emulsions.

The corresponding phase responses exhibit a similarly strong dependence on water holdup. With increasing water content, the phase slope of S_21_ becomes steeper across the frequency band, consistent with an increase in the phase constant due to a higher effective dielectric constant. The phase of S_11_ shifts toward more negative values, indicating a progressively more capacitive input impedance. These broadband results demonstrate that both magnitude- and phase-based features provide effective indicators of water holdup under homogeneous emulsion conditions.

To further evaluate the feasibility of practical sensing, representative single-frequency features are extracted from the broadband responses. As shown in Figure 8e,f, both the magnitude and phase of S_11_ and S_21_ exhibit clear and monotonic dependence on water holdup at selected frequency points. The distinct separation between curves corresponding to different water holdup levels confirms that reliable water holdup estimation can be achieved using single-frequency measurements.

**Figure 9:**
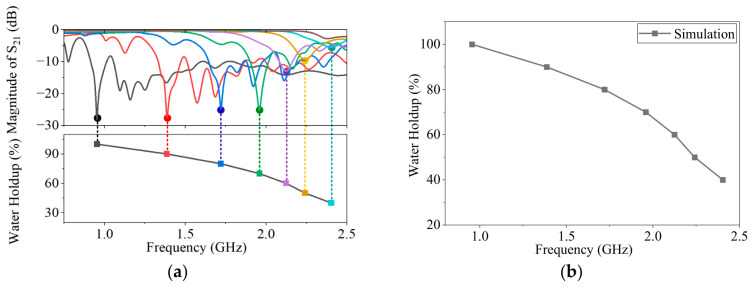
Resonant-frequency extraction under emulsion conditions. (**a**) magnitude of S_21_, (**b**) resonant-frequency in water holdup. The colors in this figure are the same as those shown in Figure 8.

In addition to broadband and single-frequency characteristics, a pronounced resonance behavior is observed in the transmission response under emulsion conditions, as highlighted in Figure 9. The resonance points are identified from the magnitude of S_21_ by tracking the dominant resonant notch/peak within the operating band, providing a well-defined frequency-domain feature that is less susceptible to amplitude scaling and phase unwrapping uncertainties. As the water holdup increases, the resonant frequency exhibits a clear, systematic shift toward lower frequencies. This trend is physically consistent with the resonance condition of a guided-wave structure: higher water content leads to a larger effective dielectric constant and higher dielectric loading around the microstrip, which increases the phase constant and effectively enlarges the electrical length of the curved transmission path. Consequently, the phase-accumulation condition for resonance is satisfied at a lower excitation frequency, resulting in a downward frequency shift.

(2) **Simulation Results for Stratified Flow**. To further evaluate the sensor performance under non-homogeneous dielectric conditions, stratified oil–water flow is considered. As shown in Figure 7b, the oil and water phases are spatially separated, resulting in an asymmetric dielectric distribution around the curved microstrip transmission line. The interface position between oil and water is varied to represent different water holdup levels while keeping the total liquid height constant.

**Figure 10:**
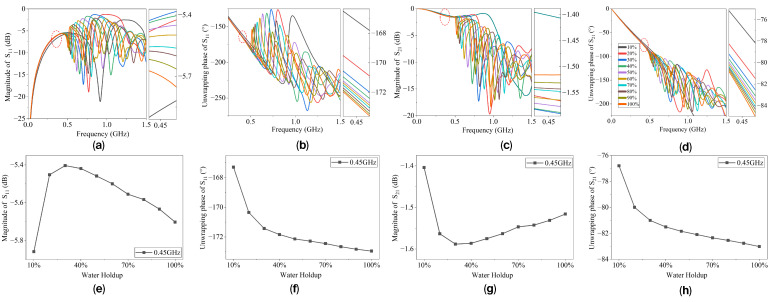
Simulated broadband responses of the sensor under stratified flow: (**a**) magnitude of S_11_, (**b**) unwrapping phase of S_11_, (**c**) magnitude of S_21_, (**d**) unwrapping phase of S_21_, and single-frequency features under stratified flow: (**e**) magnitude of S_11_, (**f**) unwrapping phase of S_11_, (**g**) magnitude of S_21_, (**h**) unwrapping phase of S_21_. The side panels are enlarged views of the regions marked by the red circles.

Figure 10a–d presents the simulated broadband magnitude and phase responses of S_11_ and S_21_ under stratified conditions. Compared with the homogeneous emulsion case, the separation between curves corresponding to different water holdup levels is noticeably reduced. This reduced resolution can be attributed to changes in interface height, inducing relatively small variations in the spatially averaged effective permittivity. As a result, the modulation of input impedance and propagation constant is weaker than that observed in homogeneous emulsion conditions. Consequently, amplitude- and phase-based features alone provide limited discrimination capability for stratified flow, especially at low frequencies.

Nevertheless, at specific frequencies, both the magnitude and phase responses exhibit identifiable, systematic variations with water holdup, as shown in Figure 10e,f. The magnitudes of S_11_ and S_21_ vary consistently with increasing water content, and the corresponding phase responses exhibit monotonic shifts. These results confirm that conventional scattering-parameter features remain physically meaningful, although their sensitivity is reduced under non-uniform dielectric loading. 

**Figure 11:**
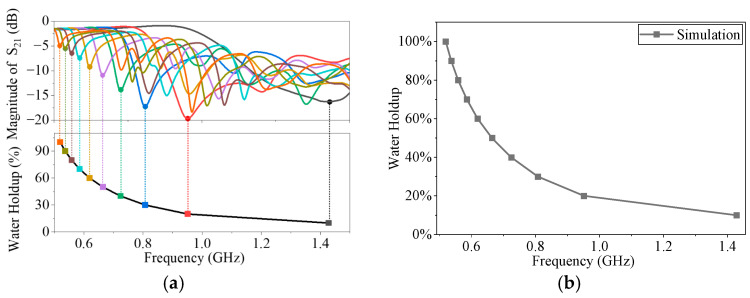
Resonant-frequency extraction under stratified oil–water conditions. (**a**) magnitude of S_21_, (**b**) resonant-frequency in water holdup. The colors in this figure are the same as those shown in Figure 10.

Despite the reduced resolution of broadband magnitude and phase features, the resonance behavior remains highly sensitive under stratified-flow conditions. As shown in Figure 11, the resonant frequency extracted from the simulated S_21_ response exhibits a clear, monotonic shift toward lower frequencies as water holdup increases. This enhanced sensitivity can be explained by the equivalent three-section transmission-line circuit model. The oil–water-loaded sensing section has a characteristic impedance and propagation constant different from those of the access and connector transition sections. The discontinuities between adjacent sections produce partial reflections and multiple wave interference along the transmission path. When the phase condition of the cascaded transmission-line system is satisfied, resonance occurs in the S-parameter response. Changes in water holdup modify $\varepsilon_{\mathrm{eff}2}$, $Z_{2}$, and $\gamma_{2}$ of the sensing section, thereby shifting the resonance frequency and changing the magnitudes of S_11_ and S_21_. As the water layer height increases, a larger fraction of the electromagnetic field interacts with the high-permittivity water region, leading to a measurable increase in effective electrical length and a corresponding downward shift in resonance frequency.

Compared with non-resonant magnitude and phase responses, the resonance frequency shift provides significantly higher resolution under stratified conditions. Although the absolute resonance frequencies differ from those observed in emulsified mixtures due to different dielectric distributions, the monotonic relationship between resonance frequency and water holdup is preserved. This confirms that resonance-based sensing serves as a dominant and robust metric for stratified oil–water flow, effectively compensating for the limited sensitivity of amplitude- and phase-based features. The numerical results demonstrate that the proposed curved microstrip transmission line sensor exhibits physically interpretable responses to variations in water holdup under non-homogeneous flow conditions. While broadband magnitude and phase features exhibit reduced separation relative to homogeneous emulsions, resonance frequency shifts remain highly sensitive and reliable. These findings highlight the importance of incorporating resonance-based sensing into the overall multi-feature framework.

## 4. Experiment Validation

To validate the proposed sensing mechanisms and numerical simulation results, experimental measurements are conducted using a fabricated prototype of the curved microstrip transmission line sensor. Experiments are conducted under both emulsion and stratified oil–water conditions to evaluate the sensor performance in homogeneous and non-homogeneous dielectric environments. The measured responses are then compared with the simulation results for trend consistency, sensitivity, and resonant behavior.

### 4.1. Sensor Fabrication

**Figure 12:**
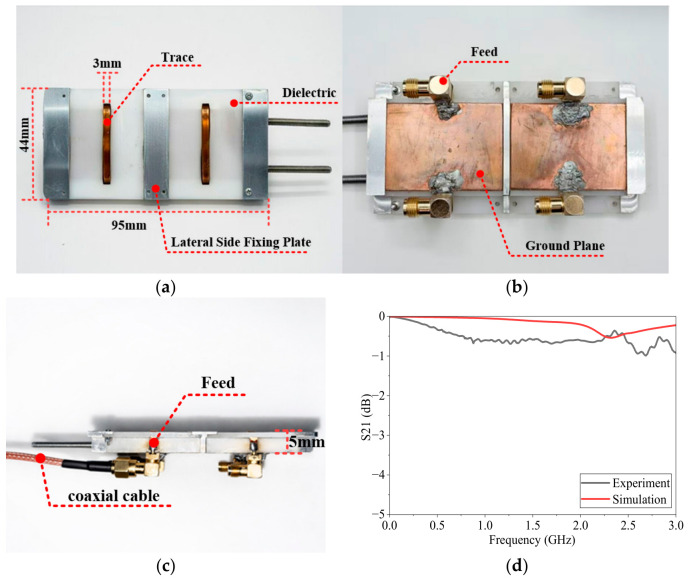
Photographs of the fabricated curved transmission-line sensor prototype and comparison of S_21_ between simulation and measurement under unloaded (air-filled) conditions: (**a**) front view of the sensor; (**b**) back view of the sensor; (**c**) side view of the sensor; and (**d**) simulated and measured S_21_ under unloaded conditions.

Figure 12 shows the photographs of the fabricated curved microstrip transmission line sensor. The sensor is manufactured with optimized geometric parameters, including the curved microstrip line, substrate, ground plane, and partitioned metal structures. The curved microstrip conductor is implemented using a flat copper strip coated with polyimide insulation, which provides electrical isolation and resistance to oil, moisture, and elevated temperatures. The substrate is fabricated from polyoxymethylene (POM) with a thickness of 1.6 mm. POM is selected for its stable dielectric properties, sufficient mechanical strength, and good machinability, making it suitable for microwave sensing applications in liquid environments. The ground plane is made of copper with a thickness of 5 mm, providing a stable reference conductor and structural support. To improve experimental reliability, two identical sensor units are fabricated. A partition metal strip is placed between the two microstrip transmission lines to suppress mutual coupling and enhance the overall structural robustness. SMA connectors with a characteristic impedance of 50 Ω are soldered to both ends of the microstrip line, providing stable, repeatable electrical interfacing with external measurement instruments.

Prior to liquid-loading experiments, baseline measurements are performed under unloaded (air-filled) conditions to characterize the intrinsic electromagnetic response of the fabricated sensor. The measured transmission coefficient S_21_ under unloaded conditions is also shown in Figure 12d. The response exhibits smooth, frequency-dependent attenuation and well-defined resonant features, indicating good fabrication quality and stable signal transmission. Comparison between the unloaded experimental results and the corresponding simulation results shows good agreement in overall trends and resonance locations. Minor discrepancies are attributed to fabrication tolerances, connector parasitics, and unavoidable assembly imperfections.

To further quantify the simulation–measurement consistency under the unloaded air-filled condition, the pointwise deviation was calculated as e_i_ = S_21_,meas(f_i_) − S_21_,sim(f_i_), where S_21_ is expressed in dB. Over the frequency range of 1 MHz–3 GHz, the mean absolute error, root-mean-square error, and maximum absolute deviation were 0.374 dB, 0.417 dB, and 0.702 dB, respectively. The maximum deviation occurred at 3.000 GHz. These results indicate that the fabricated sensor shows good consistency with the electromagnetic simulation under the unloaded condition. The remaining deviations are mainly attributed to fabrication tolerances, SMA connector parasitics, soldering effects, and assembly imperfections.

### 4.2. Experimental Setup

**Figure 13:**
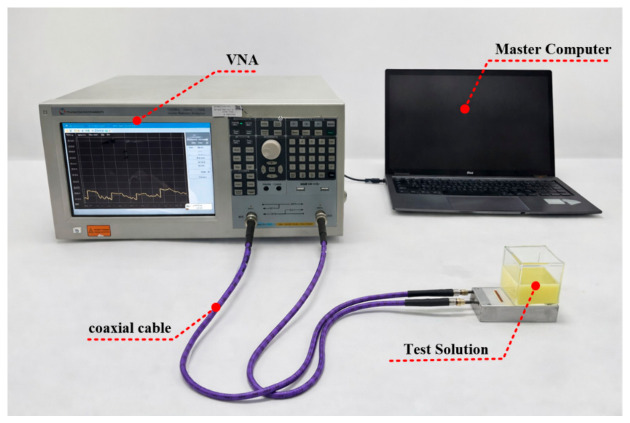
Photograph of the experimental measurement system.

The experimental setup for oil–water flow measurements is illustrated in Figure 13. The test container was an acrylic box with a length and width of 50 mm and a height of 55 mm. A TRANSCOM INSTRUMENTS T5230A vector network analyzer was used to measure the scattering parameters of the sensor over the frequency band of 1 MHz–3 GHz. The coaxial probe used in the measurement had a diameter of 3 mm. Prior to measurements, the VNA and coaxial cables were calibrated to minimize systematic errors and improve measurement repeatability.

(1) **Emulsion Oil–Water Mixtures**. Oil–water mixtures with water holdup ranging from 0% to 100% are prepared by mixing oil and water in controlled proportions, then adding an emulsifier. The mixtures are stirred sufficiently to ensure homogeneous emulsification. Each prepared emulsion is introduced into the sensing container above the sensor, with the liquid height maintained at 50 mm. After a short stabilization period, S_11_ and S_21_ (both magnitude and phase) are recorded. For each holdup condition, the measurement is repeated three times and averaged to ensure repeatability and reduce random uncertainty.

(2) **Stratified Oil–Water Flow.** For stratified experiments, water and oil are introduced sequentially into the container to form a layered configuration, with water occupying the lower region and oil the upper region. The total liquid height is kept constant at 50 mm, while the interface position is adjusted to represent different water holdup levels. After allowing the interface to stabilize, scattering-parameter measurements are performed following the same procedure as in the emulsion experiments. This experimental procedure enables systematic evaluation of the sensor response under both homogeneous and non-homogeneous dielectric distributions, providing a reliable dataset for subsequent analysis of magnitude-, phase-, and resonance-based sensing characteristics. For practical water holdup estimation, the measured S-parameter features should be interpreted through calibration rather than by directly using the ideal simulation results. In this study, the experimental measurements are used to evaluate the feasibility and response trends of the proposed sensor. In future engineering applications, reference samples or standard flow conditions with known water holdup should be used to establish calibration relationships between the measured magnitude, phase, resonance features, and water holdup. Repeated measurements, fitting residuals, mean absolute error, and root-mean-square error can then be used to evaluate the uncertainty and inversion error of the calibrated model.

### 4.3. Experimental Results and Discussion

**Figure 14:**
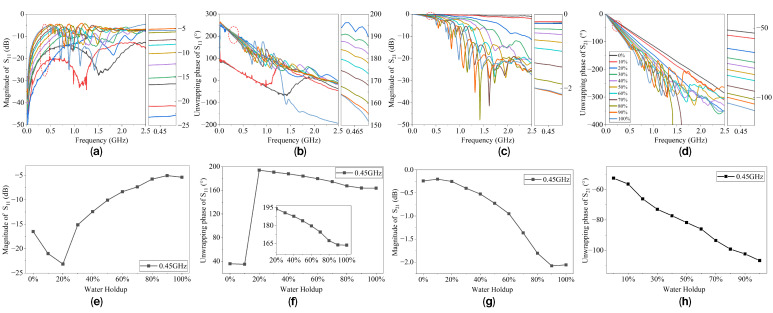
Measured broadband responses of the sensor under emulsion conditions: (**a**) magnitude of S_11_, (**b**) unwrapping phase of S_11_, (**c**) magnitude of S_21_, (**d**) unwrapping phase of S_21_, and Single-frequency features: (**e**) magnitude of S_11_, (**f**) unwrapping phase of S_11_, (**g**) magnitude of S_21_, (**h**) unwrapping phase of S_21_. The side panels are enlarged views of the regions marked by the red circles.

(1) **Emulsion Oil–Water Mixtures**. Figure 14a–d presents the measured broadband magnitude and phase responses of S_11_ and S_21_ for emulsion oil–water mixtures with varying water holdup. As the water content increases, the experimental responses exhibit clear, systematic variations that are in good agreement with numerical simulations. The magnitude of S_11_ increases progressively with water holdup, indicating an enhanced impedance mismatch due to increased dielectric loading. Meanwhile, the magnitude of S_21_ decreases monotonically due to stronger propagation attenuation associated with higher dielectric loss. The phase responses also show a strong dependence on water holdup. The phase slope of S_21_ becomes steeper as water content increases, reflecting greater phase accumulation along the transmission path. The phase of S_11_ shifts toward more negative values, consistent with a progressively more capacitive input impedance. Overall, the experimental broadband responses reproduce the trends predicted by simulation and theoretical analysis.

Representative single-frequency features are extracted from the broadband experimental data, as shown in Figure 14e,f. Both the magnitude and phase of S_11_ and S_21_ exhibit monotonic dependence on water holdup at selected frequency points, confirming the feasibility of practical water holdup estimation using simplified measurement schemes. Although the absolute separation between curves is smaller than in simulations due to additional losses and fabrication tolerances, the overall trends remain consistent.

**Figure 15:**
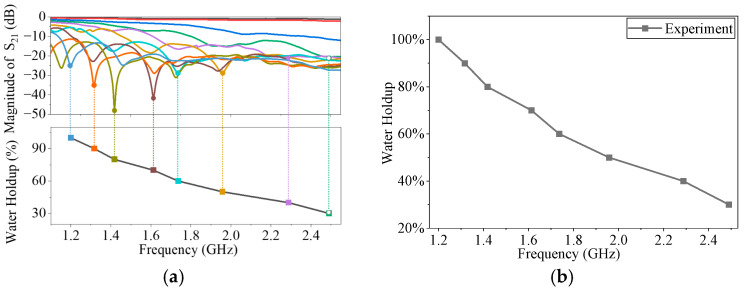
Comparison between measured and simulated resonance frequencies under emulsion oil–water. (**a**) magnitude of S_21_, (**b**) resonant-frequency in water holdup. The colors in this figure are the same as those shown in Figure 14.

In addition to broadband and single-frequency characteristics, pronounced resonance behavior is observed under emulsion conditions, as shown in Figure 15. As the water holdup increases, the resonance frequency shifts systematically toward lower frequencies. This trend agrees well with simulation results and reflects the increase in effective dielectric loading and electrical length. Under emulsion conditions, amplitude, phase, and resonance features provide mutually consistent sensing information, enabling robust multi-information characterization of water holdup.

It should be noted that, in the low-water-holdup range below 20%, the discrepancy between the experimental and simulation results becomes more noticeable. For the emulsified state, this is mainly related to the weak dielectric perturbation of low-water-holdup emulsions and the deviation of the actual sensor impedance state from the ideal simulation model. According to the dielectric parameters measured using the coaxial probe, the permittivity variation in the emulsion is relatively small within the 0–20% water holdup range. Therefore, the perturbation of the oil–water mixture to the effective permittivity and propagation characteristics of the sensor is weak, and the corresponding response variation is also small. Under this condition, fabrication tolerances, soldering-induced parasitic effects, connector losses, installation deviations, and measurement noise have a more pronounced influence on the measured S-parameter responses. This does not indicate a fundamental difficulty in detecting low water holdup, but suggests that this range is more sensitive to impedance matching and experimental uncertainties. In this range, phase information can serve as a useful auxiliary feature together with magnitude and resonance features.

To further quantify the resonance response under emulsion conditions, the experimental interval-averaged resonance-shift sensitivity was calculated from the measured resonance-frequency data. Since the resonance frequency does not vary linearly with water holdup over the entire range, a single global sensitivity cannot adequately describe the response characteristics. Therefore, the water-holdup range was divided into several approximately linear intervals, and the average resonance-shift sensitivity in each interval was calculated as $S_{f}=\frac{\left|\Delta f_{r}\right|}{\Delta H_{w}}$, where $\left|\Delta f_{r}\right|$ is the measured resonance frequency and $\Delta H_{w}$ is the water holdup. The corresponding results are summarized in Table 3.

**Figure 16:**
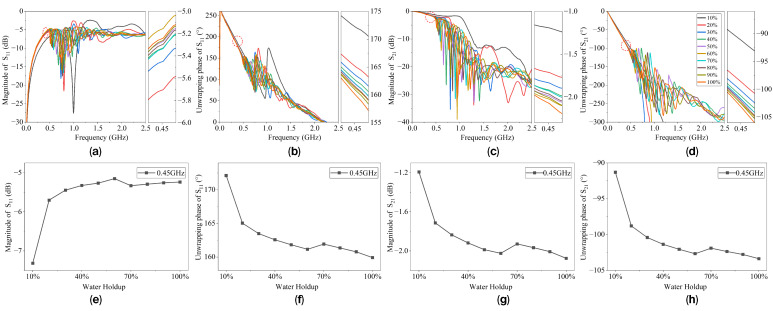
Measured broadband responses of the sensor under stratified conditions: (**a**) magnitude of S_11_, (**b**) unwrapping phase of S_11_, (**c**) magnitude of S_21_, (**d**) unwrapping phase of S_21_, and single-frequency features: (**e**) magnitude of S_11_, (**f**) unwrapping phase of S_11_, (**g**) magnitude of S_21_, (**h**) unwrapping phase of S_21_. The side panels are enlarged views of the regions marked by the red circles.

(2) **Experimental Results for Stratified Flow**. Figure 16a–d shows the measured broadband S_11_ and S_21_ magnitude and phase responses for stratified oil–water flow with different interface heights. Compared with emulsified mixtures, the broadband curves under stratified conditions exhibit increased spectral complexity due to the non-uniform dielectric distribution around the microstrip transmission line.

The magnitude responses of S_11_ and S_21_ show relatively little separation among different water holdup conditions. This indicates that, under stratified flow, changes in interface height result in weaker modulation of the equivalent impedance and attenuation than in the homogeneous emulsion case. Consequently, the amplitude-based resolution is reduced. Similarly, the broadband phase responses show consistent overall trends with increasing water holdup, but the separation between phase curves is noticeably smaller than that observed for emulsions. This reduced phase sensitivity can be attributed to the limited change in effective permittivity resulting from incremental variations in liquid height, since only part of the electromagnetic field is redistributed from air into the liquid.

Figure 16e,f presents the single-frequency magnitude and phase responses for stratified flow. Although monotonic trends in water holdup can still be identified, both amplitude and phase features exhibit lower resolution than in the emulsion case. This confirms that, under stratified conditions, conventional single-frequency magnitude- or phase-based sensing becomes less effective due to the relatively small dielectric perturbation.

**Figure 17:**
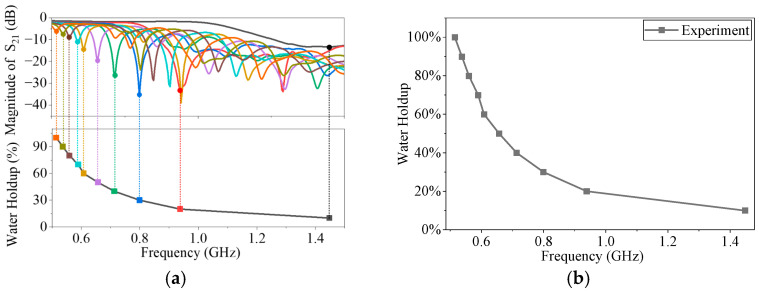
Comparison between measured and simulated resonance frequencies under stratified oil–water conditions. (**a**) magnitude of S_21_, (**b**) resonant-frequency in water holdup. The colors in this figure are the same as those shown in Figure 16.

Despite the reduced sensitivity of amplitude and phase features, the resonance behavior remains highly sensitive under stratified conditions. As shown in Figure 17, the measured resonance frequency exhibits a clear and systematic downward shift with increasing water holdup, consistent with simulation results and theoretical predictions. This observation highlights a key advantage of resonance-based sensing: even when the broadband magnitude and phase responses have limited resolution, the resonance frequency remains highly sensitive to small changes in the effective permittivity. In the stratified case, changes in interface height redistribute the electric-field energy between air and liquid regions, thereby modifying the spatially averaged effective permittivity along the curved transmission path and producing a measurable resonance shift. The resonant frequency exhibits enhanced sensitivity because it is governed by an integral phase condition over the entire guided path. Even modest dielectric perturbations can therefore generate observable frequency shifts, providing a robust and highly discriminative indicator for characterizing water holdup.

For the stratified-flow state, the response mechanism in the low-water-holdup range is different from that in the emulsified state. In stratified flow, the variation in water holdup is mainly reflected by the change in the oil–water interface position, which further affects the electric-field energy distribution near the sensor, the effective permittivity, and the equivalent electrical length of the transmission line. Compared with low-frequency magnitude and phase features, the resonance frequency is more sensitive to changes in the equivalent electrical length and interface position. Therefore, the discrepancy between the experimental and simulation results in the low-water-holdup range does not indicate a fundamental difficulty of the proposed method. Instead, it suggests that different feature combinations with different weights should be adopted for different flow states. For the emulsified state, magnitude, phase, and resonance features can be jointly used; for the stratified-flow state, more attention should be paid to the resonance frequency, while magnitude and phase can be used as auxiliary features.

For the stratified-flow case, the experimental interval-averaged resonance-shift sensitivity was calculated using the same method as that used for the emulsion case. Because the resonance frequency also varies nonlinearly with water holdup, several approximately linear intervals were selected for sensitivity evaluation. The results are summarized in Table 4 and further confirm that resonance frequency provides an effective quantitative feature for stratified-flow water holdup characterization.

The results demonstrate that the sensing behavior of the proposed curved microstrip transmission-line sensor is fundamentally governed by dielectric-loading-induced modulation of the distributed electromagnetic fields. Under homogeneous emulsion conditions, a uniform variation in effective permittivity coherently modifies the impedance, propagation constant, attenuation, and resonance frequency, leading to consistent trends in magnitude, phase, and resonance responses. In contrast, stratified flow introduces non-uniform dielectric loading, which weakens conventional magnitude- and phase-based sensitivity due to the limited changes in the spatially averaged permittivity. However, the resonant frequency remains highly sensitive because it is determined by the accumulated phase along the entire transmission path. Even small dielectric redistributions alter the effective electrical length and produce measurable frequency shifts.

**Figure 18:**
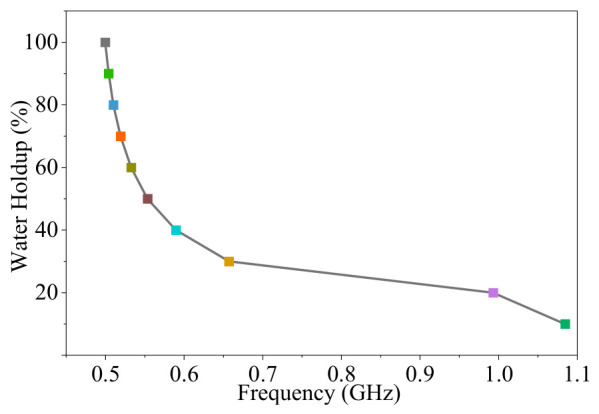
Resonance-frequency variation extracted from the S_21_ response under stratified oil–water conditions with a total liquid height of 70 mm.

For the two-layer stratified configuration, the total liquid height also affects the absolute position of the oil–water interface and the spatial distribution of dielectric loading around the sensor. Therefore, even at the same water holdup, a different total liquid height may lead to different S-parameter responses and resonance-frequency values. To examine this effect, additional simulations were performed with the total liquid height increased from 50 mm to 70 mm, while the sensor geometry, material parameters, and other simulation settings were kept unchanged. The resonance-frequency variation extracted from the S21 response under stratified oil–water conditions with a total liquid height of 70 mm is shown in Figure 18. The results show that changing the total liquid height modifies the specific response–water-holdup relationship, while the S21 responses still exhibit identifiable resonance variations with water holdup. This indicates that the proposed method remains feasible for stratified-flow sensing; however, the calibration relationship between the resonance response and water holdup should be re-established when the pipe size, liquid height, or installation condition changes.

The discrepancies mainly arise from fabrication tolerances, soldering-induced parasitic effects, connector losses, installation deviations, uncertainties in material parameters, and the idealized assumptions used in the simulation model. Therefore, for practical deployment, the sensor should be calibrated under the target pipe geometry, liquid-height range, installation condition, and operating environment. Overall, resonance-based sensing provides a structurally enhanced indicator of water holdup under the investigated emulsified and stratified conditions, while broadband magnitude and phase features offer complementary information under homogeneous emulsion conditions. The curved microstrip transmission-line architecture therefore provides a feasible sensing strategy for water holdup characterization under both homogeneous and stratified dielectric-loading conditions.

## 5. Conclusions

This study presents a curved microstrip transmission-line microwave sensor for oil–water flow characterization and establishes a systematic material–structure–response framework through dielectric measurement, full-wave simulation, and experimental validation. By incorporating experimentally measured frequency-dependent permittivity into electromagnetic modeling, the proposed approach ensures physical consistency between the dielectric properties of oil–water mixtures and the sensor-level electromagnetic responses, providing a reliable basis for mechanism analysis. The results demonstrate that variations in water holdup modulate the impedance, propagation constant, attenuation, and resonant behavior via distributed dielectric loading along the curved transmission path. Under homogeneous emulsion conditions, the broadband magnitude, phase slope, and resonance frequency exhibit coherent, monotonic trends, forming a multi-observable sensing scheme with internal redundancy and improved robustness. When the dielectric distribution becomes spatially non-uniform in stratified flow, the sensitivity of conventional magnitude- and phase-based features decreases because the spatially averaged effective permittivity changes only slightly. However, resonance frequency remains highly responsive because it is governed by the accumulated phase condition along the entire guided path. This mechanism enables resonance shift to act as a structurally enhanced and reliable indicator across different flow regimes, highlighting the intrinsic advantage of the curved microstrip transmission-line architecture. Experimental measurements confirm the theoretical and numerical predictions under the investigated emulsified and stratified conditions, demonstrating consistent response trends and validating the proposed sensing mechanism. It should be noted that the two-layer stratified model used in this study represents a fundamental and controllable case for evaluating the sensor response to oil–water interface variations. Although this model can capture the basic dielectric distribution characteristics of stable stratified oil–water flow, practical pipeline flows may involve more complex conditions, such as wavy interfaces, local emulsification, droplet entrainment, and flow-pattern transitions. Therefore, the present results should be regarded as a feasibility verification and mechanism analysis under representative fundamental flow states. The curved microstrip transmission-line design, therefore, provides an effective strategy for water holdup detection and oil–water dielectric characterization under both uniform and asymmetric loading conditions. In future work, complex flow-pattern measurements, flow-transition characterization, and systematic calibration, error-correction, and data-inversion approaches will be developed to quantitatively map combined magnitude, phase, and resonance features to water holdup in real-time pipe-flow applications.

## Figures and Tables

**Table 1:** Optimized geometrical parameters of the curved transmission line sensor (mm).

*W_a_*	*W_b_*	*W_s_*	*L_s_*	*H_a_*	*H_b_*	*H_s_*	*H_g_*
3.0	4.0	44.0	40.0	1.0	1.4	2.0	5.0

**Table 2:** Effective Cole–Cole fitting parameters of oil–water mixtures at different water holdups.

Water Holdup (%)	$\varepsilon_{s}$	$\varepsilon_{\infty }$	τ (s)	α	σ (S/m)	RMSE Real	RMSE Image
20	4.487	1.00	6.645 × 10^−12^	0.770	7.759 × 10^−5^	0.050	0.027
40	9.942	1.00	5.108 × 10^−12^	0.325	2.715 × 10^−3^	0.630	0.483
60	23.344	1.00	1.070 × 10^−11^	0.000	7.387 × 10^−3^	0.674	0.230
80	23.806	1.00	4.222 × 10^−12^	0.253	5.285 × 10^−3^	0.481	0.176
100	74.468	1.00	7.550 × 10^−12^	0.024	1.823 × 10^−20^	0.687	0.543

**Table 3:** Experimental interval-averaged resonance-shift sensitivity under emulsion conditions.

Water Holdup Interval	Resonance-Frequency Shift	Sensitivity (MHz/%)
100–80%	217.43 MHz	10.87 MHz/%
80–60%	318.64 MHz	15.93 MHz/%
60–30%	753.5 MHz	25.12 MHz/%

**Table 4:** Experimental interval-averaged resonance-shift sensitivity under stratified-flow conditions.

Water Holdup Interval	Resonance-Frequency Shift	Sensitivity (MHz/%)
100–60%	93.72 MHz	2.343 MHz/%
60–40%	104.97 MHz	5.25 MHz/%
40–20%	224.92 MHz	11.25 MHz/%
20–10%	509.83 MHz	50.98 MHz/%

## Data Availability

The data provided in this study can be obtained from the corresponding author, due to commercial restrictions.
